# Investigation of Summer Learning Loss in the UK—Implications for Holiday Club Provision

**DOI:** 10.3389/fpubh.2017.00270

**Published:** 2017-10-06

**Authors:** Jackie Shinwell, Margaret Anne Defeyter

**Affiliations:** ^1^Department of Psychology, Northumbria University, Newcastle upon Tyne, United Kingdom

**Keywords:** holiday hunger, disadvantaged communities, educational attainment, summer learning loss, school intervention

## Abstract

This study sought to examine whether summer learning loss occurs in spelling and word reading in a population of 77 primary school aged children aged between 5 and 10 years (37 boys, mean age 100 months, SD 18 months, and 40 girls mean age 103 months, SD 16 months) attending three schools in areas of low socioeconomic status in Scotland and the North East of England. Word reading and spelling was measured using the word reading and spelling subtests of the Wide Ranging Achievement Test. Participants were tested on three occassions: immediately before and immediately after a 7-week summer break, and again after 7-weeks of teaching. The results showed a significant main effect of time for spelling scores, *F*(2,136) = 21.60, *p* < 0.001, $\eta_{p}^{2}=0.241$. Post-hoc analysis [*t*(73) = 4.84, *p* ≤ 0.001] showed that spelling scores were significantly higher at the end of the summer term (M = 26.57) than at the start of the new academic year (M = 25.38). Likewise, spelling scores after 7 weeks post return to school (M = 27.61) were significantly higher than at the start of the Autumn term, *t*(73) = 7.79, *p* ≤ 0.001. Performance in spelling declined when children returned to school immediately after the summer holiday (M = 25.38) but 7 weeks later, performance had improved beyond the baseline reported immediately before the summer break (M = 26.57) [*t*(73) = 4.40, *p* ≤ 0.001]. There was also a main effect of school in relation to spelling scores, [*F*(2,68) = 6.49, *p* < 0.05, $\eta_{p}^{2}=0.160$], with children from school 2 and school 3 outperforming children from school 1. There was no signficant main effect of gender [*F*(1,68) = 1.47, *p* > 0.05, $\eta_{p}^{2}=0.021$]. None of the interactions were significant. There was no main effect of time on word reading scores [*F*(2,136) = 1.12, *p* ≥ 0.05, $\eta_{p}^{2}=0.016$]. However, there was a main effect of school [*F*(2,68) = 4.85, *p* ≤ 0.01, $\eta_{p}^{2}=0.125$] in relation to reading scores, with children from school 2 and school 3 outperforming children from school 1. There was no significant main effect of gender [*F*(1,68) = 0.37, *p* ≥ 0.05, $\eta_{p}^{2}=0.005$]. None of the interactions were significant. This is the first such study in the UK to demonstrate that after a summer break of seven weeks, summer learning loss occurred, or at least stagnation in learning, in a population of primary school aged children attending schools in areas of low SES in relation to spelling. However, after seven weeks of teaching, children caught up to and exceeded the level achieved in spelling prior to the summer break. However, the summer holiday period did not result in a loss of word reading skill, reading scores were consistent across the entire study.

## Introduction

Pupils in America spend, on average, 180 days in school receiving academic instruction whereas pupils in local authority maintained schools in the UK receive 190 days tuition per academic year (1–5). However, in America, the summer vacation is approximately 12 weeks long (5, 6), while summer holidays in the UK state education sector are typically 6–7 weeks long (4).

A conservative estimate of the effect of the USA 12-week long summer vacation on students’ standardized test scores, is that students’ learning at best stagnates, or, worst case scenario, losses of up to 1 month of grade-level equivalent learning occurs (7). Furthermore, the long summer vacation creates a gap of approximately 3 months in achievement between children from high and low socioeconomic status (SES) households (7). This gap has been referred to variously as “summer slide,” “back slide,” or “summer learning loss” (6, 8, 9) and has been studied extensively in the USA and to a lesser extent in Europe (9, 10).

Although others had investigated seasonal differences in learning, Heyns’ (11) “Summer Learning and the Effects of Schooling” is considered a seminal piece of work because it highlighted what happened to children’s learning over summer when school was not a factor in any learning, enabling an examination of the effect of “non-school” factors on levels of achievement (6, 10, 12). Heyns concluded that of the “non-school” factors examined, learning during summer was negatively related to race and income: children from low SES families tended to lose skills and knowledge, while higher SES children gained (11), a finding that has been echoed in subsequent research (1, 6, 12–14). An exception can, however, be found in a Swedish study that found no effect on students’ learning for parental SES (15).

Regardless of SES, all children show losses in maths computation and spelling skills (7, 9, 14–17). Losses in maths equate to around 1.8 months of lost learning, and losses in spelling equate to approximately 4 months’ loss of skills. A meta-analysis showed that higher SES children gained in reading while lower SES children lost skills in reading but both groups lost skills in comprehension, with lower SES children losing more (7) or, at best, tread water, making gains some summers and losing in others (1, 6, 18). However, other studies have suggested that, although higher SES children gained reading skills over the summer, the difference between them and their lower SES peers was not statistically significant and, therefore, overall, no gains were made in reading but access to reading materials, trips to the library and parental involvement in summer reading activities, and whether students had had a stimulating summer may contribute to improvements (8, 9, 12, 13).

Children from lower SES families in the USA start their academic career behind their peers from higher SES families and the educational gap in attainment continues to grow with each successive summer, regardless of the age of the pupil (6, 7, 10, 12, 14, 18). The repercussions of differing levels of achievement before even starting school, combined with successive summers of loss may compound the educational attainment gap between children of high and low SES, and this gap in achievement reverberates, not just throughout their educational career but throughout the life course of pupils (1, 19).

Similarly, evidence shows that children from low SES families in Britain start their educational career behind their peers from high SES families. The gap is evident at age three and widens by the age of five and expands at a faster rate in primary school (5–11 years) than secondary school, but poorer children still go on to perform less well in GCSE examinations at age 16 compared to children from higher SES families (20–23). Good grades in GCSE exams are strong predictors of post 16 destinations (23). As with American children, this impacts on their life course and increased likelihood of following a lower level academic route at post 16 years of age and decreased likelihood of attending university and ergo poorer employment prospects (21, 23).

Once in school, children in the USA learn at approximately the same rate regardless of SES status, and lower SES children may in fact learn at a slightly higher rate (1, 6, 8, 11, 12, 14, 17). Schooling, rather than reinforcing and entrenching differences in equality, actually serves to equalize differences in attainment by children from different SES backgrounds and stops the gap widening (1, 6, 12). Downey et al. (12) strongly assert that because children spend more time out of school than in school, the non-school environment is the biggest driver of the difference in achievement between children of different SES—it enables higher SES children to pull ahead, but school enables their more disadvantaged counterparts to catch up and reduces the rate of inequality. However, a more recent analysis of the Early Years Childhood Study, Kindergarten Class of 2010, suggests that the equalizing effect of schooling may not be maintained consistently and could even be reversed. A possible explanation could be because children from lower SES backgrounds do not have the same starting point as higher SES children and make more rapid gains in their early educational career (24). During term time, the home learning environment is also considered to be a factor contributing to widening the achievement gap between British children (20, 22). Low SES, parental level of education in low-income families, lack of parental involvement and engagement in children’s school progress and not setting high expectations are thought to contribute to an environment that is less conducive to learning, as well as a lack of access to computers, books, and academically enriching opportunities (20, 22).

A limitation of the USA-based studies relates to the testing regime, which, because data were collected in the fall and the spring, is not a true test of summer learning loss (1, 7, 11, 13, 18). As a consequence, instructional time is included in the fall results, and spring tests do not allow for the effect of teaching up to the end of term (7). In acknowledging the testing regime timings as an issue in their research, Cooper et al. (7) suggest that their estimate of time lost may underestimate the true level of loss. More recent studies extrapolated the data in regression models to reflect results that may have been achieved had the testing taken place at the start and end of the academic year, in an endeavor to reflect a more accurate picture of learning loss over the summer (10, 12, 13).

Studies in Europe have also sought to remedy the discrepancy in testing regimes. Paechter et al. (9), for instance, tested children at three time points: immediately before and after a 9-week summer vacation, and 9 weeks later, to gauge how much students’ achievements change from the start of summer vacation to the re-start of school and 9 weeks later. Losses in maths and spelling between time points one and two were found, but by time point three, students had made up for losses and slightly exceeded levels achieved at time point one. Similarly, in Germany, a study of literacy and family reading practices, tested children four times—7 weeks before and immediately prior to the start of the 6-week holiday, immediately after the summer break, and 7 weeks later to demonstrate trajectories of learning during school and summer. Students gained skills when school was in session, but stalled or lost skills in writing and reading comprehension over the summer (8).

The majority of the research relating to summer learning loss has been undertaken in the USA, with research in Europe in its infancy (9, 10). It has been suggested that summer learning loss is exclusive to the USA, amid speculation that such dramatic decreases in skills and knowledge would not be seen elsewhere as international school calendars are structured so that holidays are shorter (5). However, European-based studies have demonstrated that although holidays are shorter in Europe, summer learning loss is a phenomenon that is relevant in the European context (9, 10, 15).

A broad conclusion that can be drawn in relation to summer learning loss is that children lose skills in maths and spelling regardless of SES. High SES children may gain skills in reading and lower SES children may lose reading skills; each summer out of school appears to compound the gap in achievement between each group of children (1, 7, 11, 12). Evidence in Britain suggests that low SES contributes to the gap in attainment during term time between low and high SES children (20, 22). However, the issue of how much learning and cognitive growth occurs over the summer, and how this relates to differences in achievement between pupils has not been examined in the UK.

Given that the phenomenon of holiday hunger has been demonstrated to exist in disadvantaged areas of the UK (25, 26), we wanted to first establish whether low SES children would demonstrate similar summer learning patterns to those tested elsewhere. Given testing constraints, this paper focusses on summer learning loss in relation to spelling and word reading.

## Materials and Methods

### Design and Participants

This study employed a within subjects design whereby all children were tested at three time points: Time 1: immediately before the 7-week summer holiday; Time 2: immediately after the 7-week summer holiday, and Time 3: 7 weeks later. A total of 77 children aged between 5–10 years (37 boys, mean age 100 months, SD 18 months, and 40 girls mean age 103 months, SD 16 months) in years 1–5 and P2–P6 from three primary schools in England and Scotland participated in this study. All schools are located in areas of high deprivation/low SES and demographic characteristics of each school are presented in Table 1.

**Table 1:** School characteristics.

	School 1	School 2	School 3
Number of pupils on school roll	414	185	246
% of pupils entitled to free school meals P4–P7 Scotland, 3–6 years England	55	55	45
**Ethnicity (%)**
White/British	67	41	95
Other	33	59	5

This study received full ethical approval from the Faculty of Life Sciences’ Research Ethics Committee at Northumbria University. Informed signed consent was obtained from the head teacher of each school in accordance with local school policies and the Code of Human Research Ethics of the British Psychological Society which, in relation to gaining consent from children in schools or other institutions, states that “where the research procedures are judged by a senior member of staff or other appropriate professional within the institution to fall within the range of usual curriculum or other institutional activities, and where a risk assessment has identified no significant risks, consent from the participants and the granting of approval and access from a senior member of school staff legally responsible for such approval can be considered sufficient” (p. 17) (27). Therefore, the head teacher of each school acted in *loco parentis* and gave written consent for children to participate in the research. Parents received an information sheet about the research and an opt out consent form for their child/children. Parents had 7 days to opt their child/children out of the research study, and four parents asked for their child to be excluded. At each stage of data collection, children received written age appropriate information to take home and were verbally asked if they wished to participate/continue their participation in the study. It was made clear that they were entitled to withdraw at any stage. At the end of the study, children were given a debrief sheet to take home. The debrief sheet reminded children/parents that they had the right to withdraw from the study. Sixteen children were withdrawn from the study due to absence, their withdrawal of consent, and experimenter error.

### Measures

Word reading and spelling was measured using the word reading and spelling subtests of the Wide Ranging Achievement Test (WRAT 4). The word reading subtest measures letter and word decoding. Part 1 consists of a list of 15 letters. Part 2 consists of a list of 55 words that increase in difficulty as the test proceeds, with a combined maximum score of 70. Participants read the letters and/or words aloud as appropriate. The spelling subtest measures an individual’s ability to encode sounds into written form from dictated letters and words and consists of two parts. Part 1, letter writing consists of name writing (2 marks) and writing 13 dictated letters. Part 2 consists of 42 words that increase in difficulty as the test proceeds, which are dictated to participants. The combined maximum score for the spelling subtest is 57.

Each test has two alternate forms (blue and green) and can be used interchangeably as pre- and post-test measures (28). Due to the interchangeability of both versions of the measure, the green version of the tests was randomly chosen and used at time 1. At time 2, the blue version of the test was used and at time 3, the green version of the test was administered. Alternate form immediate retest reliability coefficients of the WRAT 4 range from 0.78 to 0.89 for an age-based sample and from 0.86 to 0.90 for a grade-based sample. The alternate-form delayed (approximately 30 days) retest study indicates that practice effects are quite small. Mean score differences of 0.4–2.2 were found for an age-based sample; differences of 0.1–0.5 were found for a grade-based sample (28). The same letters are used in part 1 of both the green and blue versions of the word reading and spelling tests.

### Procedure

Data were collected on school premises during school hours. The researcher worked with classes/pupils that were available and not engaged in other school-based activities. A quiet area of each school was identified to work with children on a one-to-one basis with children aged less than 8 years of age to administer both the word reading and spelling subtests with each child and to administer the word reading subtest with children aged over 8 years of age. The researcher worked in class with groups of children aged over 8 years of age to administer the word spelling subtest.

All children under 8 years of age and any child over 8 years of age who achieved fewer than five correct answers in Part 2 of either the word reading test or word spelling test were required to complete Part 1 of each test. Children aged over 8 years of age who correctly achieved more than five correct answers in Part 2 of each test were automatically credited with 15 marks for Part 1 of each test. Each test was conducted until either 10 incorrect answers were achieved or the end of each test was reached (5/10 rule) (28).

In accordance with the WRAT 4 manual, the word reading test was administered first. Children taking Part 1 of the word reading test were handed a printed page containing 15 letters and was given 5 s to read each letter aloud. One mark was allocated for each correctly pronounced letter. When administering part 2 of the word reading test, the researcher handed each child a copy of the appropriate version (green or blue) of the list of words and asked each child to read each word aloud until 10 consecutive errors were made or the end of the list of words was reached. Each child was given 10 s to pronounce each word. After the first error, the child was asked to repeat the word that was miss-pronounced. If it was pronounced correctly the second time, it was scored correctly. After the first error, the participant was not given the opportunity to repeat any further words that were miss-pronounced and were, therefore, scored incorrectly.

When undertaking Part 1 of the word spelling test, each child was first asked to write their name. Two marks were awarded for two clearly identifiable as correct letters of the child’s name. Thereafter, a series of 13 letters were dictated one at a time, and each child was given 5 s to write each letter. The test proceeded until 10 consecutive errors were made or the end of the list of letters was reached. If the child did not make 10 consecutive errors in Part 1 of the test, Part 2 of the word reading test was administered. When administering Part 2 of the word reading test, the researcher read each word to be written aloud followed by a sentence containing the word. The word to be written was repeated and 15 s was allowed for each word to be written. If a participant was in the middle of writing a word when the 15 s had elapsed, they were permitted to complete the word. When working on a one to one basis, the test proceeded until either 10 consecutive errors were made or the end of the list of words was reached. In accordance with the WRAT manual, the 10 rule was waived when working with groups of children to avoid individual embarrassment. However, no marks were given for correctly spelled words after 10 consecutive errors.

### Data Analysis

Raw score data were entered IBM SPSS (v24) and three outliers were removed prior to further analyses by means of one way repeated measures ANOVA.

## Results

The mean scores and SD for spelling and reading are presented in Table 2.

**Table 2:** Means and (SD) of spelling and reading raw scores at end of summer term (T1), start of new academic year (T2), and 7 weeks post return to school (T3).

*N* 74	Time	Spelling Mean	Reading Mean
	1	26.57 (5.94)	36.43 (10.35)
	2	25.38 (5.88)	36.96 (10.35)
	3	27.61 (6.06)	36.41 (10.23)

### Spelling

The results showed a significant main effect of time for spelling scores, *F*(2,136) = 21.60, *p* < 0.001, $\eta_{p}^{2}=0.241$.

**Figure 1:**
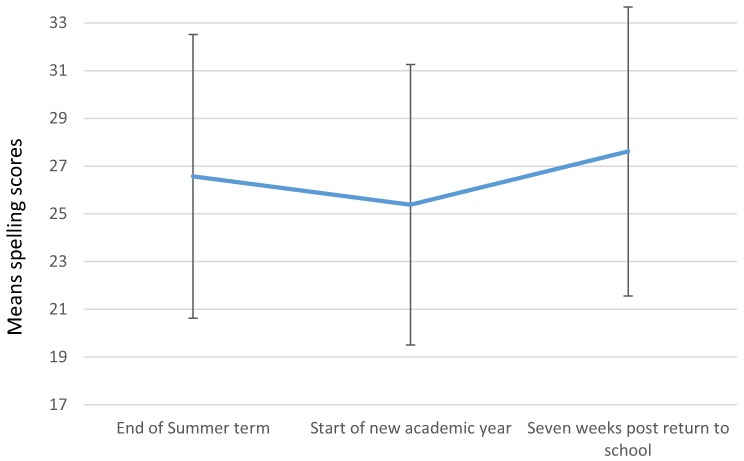
Mean spelling scores as a function of time.

*Post-hoc* analysis [*t*(73) = 4.84, *p* ≤ 0.001] showed that spelling scores were significantly higher at the end of the summer term (M = 26.57) than at the start of the new academic year (M = 25.38). Likewise, spelling scores after 7 weeks post return to school (M = 27.61) were significantly higher than at the start of the Autumn term [*t*(73) = 7.79, *p* ≤ 0.001]. (These findings are represented in Figure 1.) Performance in spelling declined when children returned to school immediately after the summer holiday (M = 25.38) but 7 weeks later, performance had improved beyond the baseline reported immediately before the summer break (M = 26.57) (*t*(73) = 4.40, *p* ≤ 0.001).

Analysis showed no significant main effect of gender [*F*(1,68) = 1.47, *p* > 0.05, $\eta_{p}^{2}=0.021$]. There were no significant interactions between gender × time [*F*(1,68) = 0.115, *p* > 0.05, $\eta_{p}^{2}=0.002$] or between gender × school [*F*(2,68) = 0.035, *p* > 0.05, $\eta_{p}^{2}=0.001$].

There was a significant main effect of school [*F*(2,68) = 6.49, *p* < 0.05, $\eta_{p}^{2}=0.160$]. However, there was no significant time × school interaction [*F*(2,68) = 0.89, *p* > 0.05, $\eta_{p}^{2}=0.026$].

*Post-hoc* analysis showed that the overall spelling scores for school 1 (M = 20.73) were significantly lower than the spelling scores for both school 2 (M = 27.12) and for school 3 (M = 27.45).

Finally, there was no significant three-way interaction between school × time × gender [*F*(2,68) = 1.38, *p* > 0.05, $\eta_{p}^{2}=0.039$].

### Reading

A repeated measures ANOVA for reading showed no significant main effect of time on word reading scores [*F*(2,136) = 1.12, *p* > 0.05, $\eta_{p}^{2}=0.016$]. Also, there was no significant main effect of gender [*F*(1,68) = 0.37, *p* > 0.05, $\eta_{p}^{2}=0.005$]. There was no significant gender × time interaction [*F*(1,68) = 0.66, *p* > 0.05, $\eta_{p}^{2}=0.010$], nor a significant gender × school interaction [*F*(2,68) = 0.19 *p* > 0.05, $\eta_{p}^{2}=0.005$].

There was a main effect of school [*F*(2,68) = 4.85, *p* < 0.01, $\eta_{p}^{2}=0.125$]. However, there was no significant time × school interaction [*F*(2,68) = 0.76 *p* > 0.05, $\eta_{p}^{2}=0.022$]. *Post-hoc* analysis showed that reading scores for school 1 (M = 27.70) were significantly lower than reading scores for both school 2 (M = 38.75) and school 3 (M = 37.86).

Finally, there was no significant three-way interaction between school × time × gender [*F*(2,68) = 0.62, *p* ≥ 0.05, $\eta_{p}^{2}=0.018$].

## Discussion

This study sought to identify whether summer learning loss occurs in spelling and word reading in a population of primary school aged children residing in areas of low SES in the North East of England and west of Scotland who did not attend a holiday club over the summer.

The results of this study showed that following a 7-week summer break, a small but significant effect of summer learning loss occurred in relation to spelling. These results accord with prior work including the meta-analysis by Cooper et al. (7), work by Allinder et al. (16) and Paechter et al. (9). It is important to note that while the loss in spelling between T1 and T2 is significant [*t*(73) = 4.84, *p* = < 0.001], there is only a small change in mean scores from M = 26.57 to M = 25.38 betweenT1 and T2. While this effect is small, the data clearly demonstrate that at the very least, learning in terms of spelling stagnates over the summer period.

Learning to spell is a complex process with many rules and exceptions relating to phonology and morphology (29), with children using a range of strategies, beginning with a simple phonetic phase but later apply more complex strategies as their knowledge of orthographic rules increases (30). Six broad spelling strategies, e.g., sounding out and retrieving words and use of rules were identified by Rittle-Johnson and Siegler (31), who, in their longitudinal study that saw children undertake spelling tests in first and second grade. The authors found that first grade children used between two and five strategies to spell, with second grade children using between two and six strategies. Cooper et al. (7) suggested that spelling skills, like maths skills, require procedural knowledge which needs to be learned and reinforced through practice, and opportunities to do so are lacking in the home environment over summer, making knowledge gained in school susceptible to loss (7).

However, this study further demonstrated that after 7 weeks of teaching, the level of achievement in spelling exceeded that which had been achieved at the end of the previous academic year. This means, as suggested by Paechter et al. (9), that children could compensate for the loss of spelling skills over the summer and then go on to increase the level of skill after 7 weeks of teaching, but the first few weeks of school are negatively affected by learning loss as children first have to catch up to accommodate lost knowledge and skills. This finding, therefore, has important implications in terms of demonstrating that, even after a break some 5 weeks shorter than their American counterparts, UK children lose skills in spelling. Thus, at the start of the new academic year, children must first regain lost skills and knowledge before progressing.

The results of this study also demonstrated that summer learning loss did not occur in relation to word reading which is contrary to the findings of Cooper et al. (7) whereby children from low SES families lost skills in reading. The analysis of the reading comprehension component of the California Achievement Test suggested that children from low SES families lost skills and knowledge over the summer, perhaps gaining or losing in some years but effectively treading water, while higher SES children gained (1, 6). However, some studies report no gains in literacy (8, 12, 13). A reason for this difference in reported results between studies could quite simply be due to lack of homogeneity in the measures used and different aspects of reading skill were being measured. Downey et al. (12) and Burkam et al. (13) may have been using measures that broadly sought to assess more technical aspects of reading rather than the broader “reading comprehension” used by Alexander et al. in their analysis. Downey et al. (12) measured upper and lower case letter recognition, word sounds at the beginning and end of words and word recognition by sight. Burkam et al. (13) measured print familiarity, letter recognition, beginning and ending word sounds, rhyming words, word recognition, vocabulary, and comprehension. These aspects of reading may have more similarities to the skills used in word decoding measured by the WRAT 4, making the findings of Burkam et al. (13) and Downey et al. (12) more relevant to this study. An ability to decode enables learners to recognize and read words they have never encountered before (32). Word decoding has been described as a continuum or trajectory that ranges from slow and laborious to rapid and effortless decoding of words that becomes automatic as readers gain experience (33). It may simply be that children did not lose skills in word reading over the summer due to the fact that they can continue to read words as part of everyday life, which, unlike spelling, does not require continued practice and reinforcement to master. However, this does not explain why, after 7 weeks of teaching, children’s level of achievement in word reading in this study did not improve. However, it may be that it takes longer for evidence of improvement in skills in word reading to be demonstrated than the time frame used in this study, although it should further be noted that the word reading element of the WRAT 4 (which measures letter and word decoding) was found to be a strong valid proxy measure for education quality and a key predictor of neurocognitive performance (34).

This study further found no effect of gender in relation to word reading or spelling. This is consistent with the findings of Cooper et al. (7) in their meta- analysis, but contrary to the observed trend of girls out performing boys in reading in international studies and studies relating to learning loss, with the gap by gender being apparent before children start school and during school, whereby girls learn at a faster rate in school (9, 12, 17, 35–37). More broadly, in the UK, boys of secondary school age who are entitled to free school meals make less progress at school than girls entitled to free school meals (22).

This study further demonstrated an effect of school, with school 2 and school 3 outperforming school 1 for both spelling and reading. However, while there was a significant main effect of school it is important to note that there were no significant school × time interactions.

Although holiday clubs and summer schools are well established in the USA (38), the need for some form of provision within the UK to address the phenomenon of holiday hunger, whereby children who would normally access free school meals during term time has come to the fore recently (25, 26). The potential opportunity to provide enrichment activities for children has also been suggested. This study provides preliminary findings suggesting that holiday clubs may be an effective means of providing children with educational activities across the summer, which in turn may alleviate any drop or stagnation in educational performance.

In summary, this study is the first UK-based study to demonstrate that that summer learning loss, or at least stagnation, occurs in a population of children attending schools in areas of low SES in relation to spelling, but that after 7 weeks of teaching, children were able to exceed the level they achieved prior to the summer holiday. However, the summer holidays did not result in a loss of word reading skills. Although children maintained and did not lose skills in reading over the summer, the results of this study suggest that, unlike spelling, they did not make any achievement after 7 weeks of teaching.

However, this study is not without limitations. First and foremost, this study did not assess whether summer learning loss occurs in relation to maths skills. Additionally, a further limitation relates to the study sample, which only included children from areas of low SES. Research in the USA and Europe suggests that skills are lost in maths and that where higher SES children gained skills in reading, this drives the gap in achievement between each socioeconomic group throughout their educational and post-education destinations. Awareness of whether children lose skills in maths and whether children from higher SES families gain or lose skills in the two domains tested in this study and maths could have important implications for UK educational policy and further inform the need for the type and scope of holiday provision in the UK. Future work will address these limitations.

## Ethics Statement

This study was carried out in accordance with the recommendations of the Research Ethics and Governance Handbook of Northumbria University with written informed consent from all subjects. All subjects gave written informed consent in accordance with the Declaration of Helsinki. The protocol was approved by the Faculty of Life Sciences’ Research Ethics Committee at Northumbria University.

## Author Contributions

JS was involved in the design, data collection and analysis of data, and writing this research. MD was involved in the design, analysis, and writing of this research.

## Conflict of Interest Statement

The authors declare that the research was conducted in the absence of any commercial or financial relationships that could be construed as a potential conflict of interest.
